# The occupational burnout among medical staff with high workloads after the COVID-19 and its association with anxiety and depression

**DOI:** 10.3389/fpubh.2023.1270634

**Published:** 2023-10-26

**Authors:** Hao Sun, Tengfei Zhang, Xinyu Wang, Caixia Wang, Mengqiao Zhang, Hongjiang Song

**Affiliations:** ^1^Department of Gastrointestinal Surgery, Harbin Medical University Cancer Hospital, Harbin Medical University, Harbin, Heilongjiang, China; ^2^Department of Breast Surgery, Harbin Medical University Cancer Hospital, Harbin Medical University, Harbin, Heilongjiang, China

**Keywords:** burnout, medical staff, COVID-19, Cronbach’s α and confirmatory factor analysis, anxiety, depression

## Abstract

**Objective:**

After the end of COVID-19, medical staff were immediately faced with a high workload, leading to widespread occupational burnout. This study aims to explore the level and influencing factors of burnout among medical staff during this period, as well as its relationship with anxiety and depression.

**Methods:**

The participants’ levels of burnout were assessed using Maslach Burnout Inventory-Human Services Survey (MBI-HSS), and the reliability and validity of the questionnaire were evaluated through Cronbach’s α and Confirmatory Factor Analysis (CFA). Independent sample *t*-test, chi-square test, and Pearson analysis were employed to determine the correlation between two sets of variables. Univariate and multivariate logistic regression analyses were conducted to identify significant factors influencing burnout. Finally, nomograms were used to predict the probability of burnout occurrence.

**Results:**

This study collected a total of 1,550 questionnaires, and after excluding 45 questionnaires that were duplicates or incomplete, a sample of 1,505 (97.1%) participants were included in the final statistical analysis. Both Cronbach’s α and the fit indices of CFA demonstrated excellent adaptability of the Chinese version of MBI-HSS in this study. The overall prevalence rates for emotional exhaustion (EE), depersonalization (DP), and diminished personal accomplishment (PA) were 52.4, 55.3, and 30.6%, respectively. Obtaining psychological support, health condition, relationship with family members, and insufficient sleep were identified as common contributing factors to burnout among medical staff. Additionally, age and promotion pressure were also associated with burnout among doctors, and exceeding legal working hours was an important factor for nurse burnout. The C-index for the nomograms predicting burnout among doctors and nurses was 0.832 and 0.843, respectively. Furthermore, burnout exhibited a significant linear correlation with anxiety and depression.

**Conclusion:**

After the end of COVID-19, medical staff in high workload environments were facing severe burnout, which might lead to anxiety and depression. The occupational burnout of medical staff needed to be taken seriously and actively intervened.

## 1. Introduction

Burnout is a major and prevalent public health issue, significantly impacting the physical and mental health of individuals across various industries (1). It was first introduced in 1974 and defined as a state of mental exhaustion caused by one’s professional career (2). Burnout often arises from the combination of high stress and high ideals, and its primary characteristics are emotional exhaustion and reduced work efficiency (3). Medical staff frequently face high levels of responsibility, pressure, and emotional burden in their daily work, which can lead to prolonged periods of intense work and make them susceptible to experiencing burnout symptoms (4). Numerous psychological studies targeting medical staff found higher rates of burnout among them (5, 6). The emergence of COVID-19 has significantly exacerbated this phenomenon (7, 8). As a key force in fighting against COVID-19, medical staff always face great psychological stress during COVID-19 (9). Studies have shown that medical staff experienced higher levels of occupational burnout, anxiety, depression, and other psychological issues during the COVID-19 outbreak, severely affecting their mental health (10, 11).

During the COVID-19 pandemic, various public places, including hospitals, have either been closed or implemented extremely strict preventive measures to prevent the spread of the virus (12). Indeed, this has significantly reduced the public’s willingness to seek medical care, leading to a substantial decrease in the number of visits from non-COVID-19 related patients (13–15). After the strict policies targeting COVID-19 were lifted, there was a significant increase in the number of patients in hospitals nationwide. The immense workload has placed a tremendous psychological burden on medical staff, with some working for more than 12 h a day. Despite the pandemic’s end, the burnout levels among medical staff continue to remain unexpectedly high. Therefore, it is still crucial to study the burnout, anxiety, and depression among medical staff at this stage. Identifying key factors related to burnout and developing predictive models for burnout in healthcare workers can help identify high-risk individuals and formulate appropriate psychological intervention strategies.

Maslach and Jackson summarized burnout into three dimensions: emotional exhaustion (EE), depersonalization (DP), and diminished personal accomplishment (PA) (16). In 1981, they developed the Maslach Burnout Inventory (MBI) to measure burnout levels based on these dimensions (17). Over time, the MBI has been adapted into multiple versions for assessing different populations (18, 19). This study investigated the prevalence of occupational burnout and its risk factors among medical staff after the COVID-19 using the Maslach Burnout Inventory-Human Services Survey (MBI-HSS). We also examined the adaptability and reliability of the Chinese version of the MBI-HSS. Additionally, we developed a predictive model to identify individuals at high risk of burnout. Previous research has suggested a certain correlation between anxiety, depression, and burnout (20). Considering that anxiety and depression are critical factors influencing mental well-being and may lead to severe consequences, this study further analyzed their interaction with burnout.

The research hypotheses of this study included:

*H1*: After the end of COVID-19, the sudden increase in workload has led to elevated levels of professional burnout among doctors and nurses, which is influenced by various factors.

*H2*: After the end of COVID-19, doctors and nurses still experience a certain degree of anxiety and depression.

*H3*: There is a significant correlation between burnout and anxiety and depression, with prolonged burnout leading to elevated levels of anxiety and depression.

## 2. Materials and methods

### 2.1. Participants

We conducted this multicenter cross-sectional survey from April 1 to May 31, 2023. Due to the convenience of an electronic questionnaire, we anonymously surveyed medical staff using a professional survey platform.^1^ After excluding incomplete questionnaires, a total of 1,505 medical staff from 24 provinces were included in the final analysis. These participants were primarily from Heilongjiang, accounting for 21.6% of the total, with the remainder coming from Sichuan (14.0%), Beijing (13.2%), Inner Mongolia (10.7%), Ningxia (10.2%), Jilin (9.9%), Shandong (7.5%), Zhejiang (6.5%), and other provinces (6.4%). This study was supported by the Ethics Committee of Cancer Hospital of Harbin Medical University (Ethics approval number: 2019-22-IIT).

### 2.2. Questionnaire design

The questionnaire used in this study comprised three sections, totaling 53 questions. The first section consisted of 15 items. The first 6 items gathered participants’ basic information, including sex, age, occupation, department, educational level, and marriage status. The remaining nine items explored the common factors influencing the psychological status of medical staff, as determined based on relevant previous research (21–23). This study used the MBI-HSS to assess the level of burnout among medical staff. The MBI-HSS is a specialized version of the Maslach Burnout Inventory (MBI) developed specifically for healthcare professionals, comprising three dimensions: emotional exhaustion (EE) (Nine items, including questions 1, 2, 3, 6, 8, 13, 14, 16, and 20), depersonalization (DP) (Five items, including questions 5, 10, 11, 15, and 22), and personal accomplishment (PA) (Eight items, including questions 4, 7, 9, 12, 17, 18, 19, and 21), with a total of 22 items. Considering the potential inconvenience for participants caused by the large questionnaire, the third section utilized the Generalized Anxiety Disorder Questionnaire 7 (GAD-7) and the Patient Health Questionnaire 9 (PHQ-9) to assess the participants’ anxiety and depression status. GAD-7 is an anxiety assessment scale consisting of 7 questions. It was developed by Spitzer and his colleagues in 2006 by collecting relevant information from 2,740 participants across 15 institutions. They found that GAD-7 not only has good reliability and validity, but also has the highest sensitivity and specificity when the cutoff value was 10 (24). A series of subsequent studies also confirmed their conclusion and found that GAD-7 has high adaptability in different countries and populations (25, 26). Kroenke and his colleagues first validated the effectiveness of PHQ-9 in measuring the severity of depression in 2001. Through the analysis of many samples, they discovered that PHQ-9 not only could diagnose depression but also accurately reflect the level of depression. Furthermore, PHQ-9 also achieved the highest sensitivity and specificity when the cutoff value was set at 10 (27). Subsequent research on PHQ-9 in different populations further confirmed its high adaptability (28, 29). The Chinese versions of BNI-HSS, GAD-7, and PHQ-9 could be found in the Supplementary materials.

### 2.3. Assessment of occupational burnout, anxiety, and depression

All questions in the MBI-HSS have seven options, which were scored from 0 to 6 based on severity. The total scores for EE, DP, and PA were calculated separately by summing up the scores of all questions within each respective section. According to previous studies, participants were considered to suffer from EE, DP, and diminished PA when their EE score ≥ 27, DP score ≥ 10, and PA score < 33, respectively.

Occupational burnout was determined through the scores of the three sections of MBI-HSS, and there were multiple versions of this assessment. The commonly used diagnostic criteria included two approaches: a relatively lenient standard that considers participants with EE score ≥ 27 or DP score ≥ 10 to have symptoms of burnout, and a more stringent standard that requires the participant to meet all three criteria simultaneously, including EE score ≥ 27, DP score ≥ 10, and PA score < 33, to be diagnosed with burnout (30).

All questions in the GAD-7 and PHQ-9 consisted of four items, scored from 0 to 3 based on severity. The total score for each scale was obtained by summing up the scores of all questions, and the optimal cutoff value for both GAD-7 and PHQ-9 was 10 points. In addition, the severity of anxiety and depression can be assessed based on the scores as follows: Normal (0–4), Mild (5–9), Moderate (10–14), and Severe (>15).

### 2.4. Statistical analysis

The main statistical analysis was conducted using SPSS 25.^2^ Categorical variables were presented as *n* (%) and analyzed using the chi-square test or Fisher’s exact test. The distribution of continuous variables was assessed using the one-sample Kolmogorov–Smirnov (K-S) test. Continuous variables with a Gaussian distribution were expressed as mean ± standard deviation (SD) and analyzed using the independent samples *t*-test and Pearson correlation analysis. For continuous variables not following a Gaussian distribution, median with interquartile range was used, and differences were evaluated using the Mann–Whitney U test. In addition, we conducted univariate and multivariate logistic regression analyses to identify independent factors influencing occupational burnout, presenting the results as Hazard Ratios (HR) with corresponding 95% Confidence Intervals (CI). The Multicollinearity in multivariate analysis was tested by variance expansion factor (VIF). The nomograms for predicting the probability of occupational burnout and the calibration curves for verifying the predictive performance of the nomograms were created using R 4.2.3.^3^ A two-sided *p*-value of <0.05 was considered to have a statistically significant difference.

The reliability of MBI-HSS, GAD-7, and PHQ-9 was evaluated using Cronbach’s α. The validity of the MBI-HSS was evaluated through CFA. Fit indices, including root mean square error of approximation (RMSEA), comparative fit index (CFI), and Tucker-Lewis index (TLI), were used to assess structural validity. Average variance extracted (AVE) and composite reliability (CR) scores, calculated by standardized factor loadings (SFL), were used to evaluate convergent validity. Additionally, standardized correlation coefficients between different factors were used to assess discriminant validity. All validations were conducted using Mplus 8.9^4^ and Amos 26.^5^

## 3. Results

### 3.1. Participants sample characteristics

This study collected a total of 1,550 questionnaires, and after excluding 45 questionnaires that were duplicates or incomplete, a sample of 1,505 (97.1%) participants were included in the final statistical analysis. Among them, there were 378 (25.1%) males and 1,127 (74.9%) females, with an average age of 35.77 (SD = 7.90) years. 731 participants (48.6%) were doctors, among whom 326 individuals (44.6%) work in surgery. There were 774 nurses (51.4%), with 311 individuals (40.2%) working in surgery. Additionally, over half of the doctors (58.0%) held a master’s or higher degree, while almost all nurses are female (96.8%) and almost all have undergraduate or lower education (97.4%). The correlation analysis revealed significant differences between doctors and nurses in terms of age, sex, educational level, obtaining psychological support, participation in epidemic prevention, income increase, exceeding legal working hours, promotion pressure, and insufficient sleep (all *p* < 0.05), as shown in Table 1.

**Table 1:** Participants characteristics.

Items, *n* (%)	Occupation			*P*
	Total	Doctor	Nurse	
	*n* = 1,505	*n* = 731	*n* = 774	
Age, mean (SD)	35.77 (7.90)	36.66 (8.93)	34.93 (6.67)	<0.001
Sex				<0.001
Male	378 (25.1)	353 (48.3)	25 (3.2)	
Female	1,127 (74.9)	378 (51.7)	749 (96.8)	
Department				0.083
Surgery	637 (42.3)	326 (44.6)	311 (40.2)	
Non-surgery	868 (57.7)	405 (55.4)	463 (59.8)	
Educational level				<0.001
Undergraduate or below	1,061 (70.5)	307 (42.0)	754 (97.4)	
Master or above	444 (29.5)	424 (58.0)	20 (2.6)	
Marriage status				0.087
Yes	1,141 (75.8)	540 (73.9)	601 (77.6)	
No	364 (24.2)	191 (26.1)	173 (22.4)	
Relationship with family members				0.777
Well	1,385 (92.0)	669 (91.5)	716 (92.5)	
Medium	116 (7.7)	60 (8.2)	56 (7.2)	
Poor	4 (0.3)	2 (0.3)	2 (0.3)	
Obtaining psychological support				<0.001
Yes	489 (32.5)	189 (25.9)	300 (38.8)	
No	1,016 (67.5)	542 (74.1)	474 (61.2)	
Health condition				0.249
Well	1,112 (73.9)	532 (72.8)	580 (74.9)	
Medium	349 (23.2)	181 (24.8)	168 (21.7)	
Poor	44 (2.9)	18 (2.5)	26 (3.4)	
Health condition of family members				0.257
Well	1,167 (77.5)	554 (75.8)	613 (79.2)	
Medium	315 (20.9)	166 (22.7)	149 (19.3)	
Poor	23 (1.5)	11 (1.5)	12 (1.6)	
Participation in epidemic prevention				<0.001
Yes	1,116 (74.2)	510 (69.8)	606 (78.3)	
No	389 (25.8)	221 (30.2)	168 (21.7)	
Income increase				<0.001
Yes	377 (25.0)	136 (18.6)	241 (31.1)	
No	1,128 (75.0)	595 (81.4)	533 (68.9)	
Exceeding legal working hours				<0.001
Yes	738 (49.0)	485 (66.3)	253 (32.7)	
No	767 (51.0)	246 (33.7)	521 (67.3)	
Promotion pressure				<0.001
Yes	952 (63.3)	518 (70.9)	434 (56.1)	
No	553 (36.7)	213 (29.1)	340 (43.9)	
Insufficient sleep				0.037
Yes	1,142 (75.9)	572 (78.2)	570 (73.6)	
No	363 (24.1)	159 (21.8)	204 (26.4)	

SD, standard deviation.

### 3.2. Reliability analysis

To explore reliability, we calculated the Cronbach’s α for MBI-HSS, GAD-7, and PHQ-9. The results showed that their Cronbach’s α were all within the range of 0.8 to 1, indicating a high level of internal consistency (Table 2).

**Table 2:** Reliability analysis of MBI-HSS, GAD-7, and PHQ-9.

Items	Cronbach’s α	Number of questions
EE	0.932	9
DP	0.818	5
PA	0.877	8
MBI-HSS	0.830	22
GAD-7	0.944	7
PHQ-9	0.911	9

EE, emotional exhaustion; DP, depersonalization; PA, personal accomplishment; GAD-7, Generalized Anxiety Disorder Questionnaire-7; PHQ-9, Patient Health Questionnaire-9; MBI-HSS, Maslach Burnout Inventory-Human Services Survey.

### 3.3. Validity analysis

**Figure 1:**
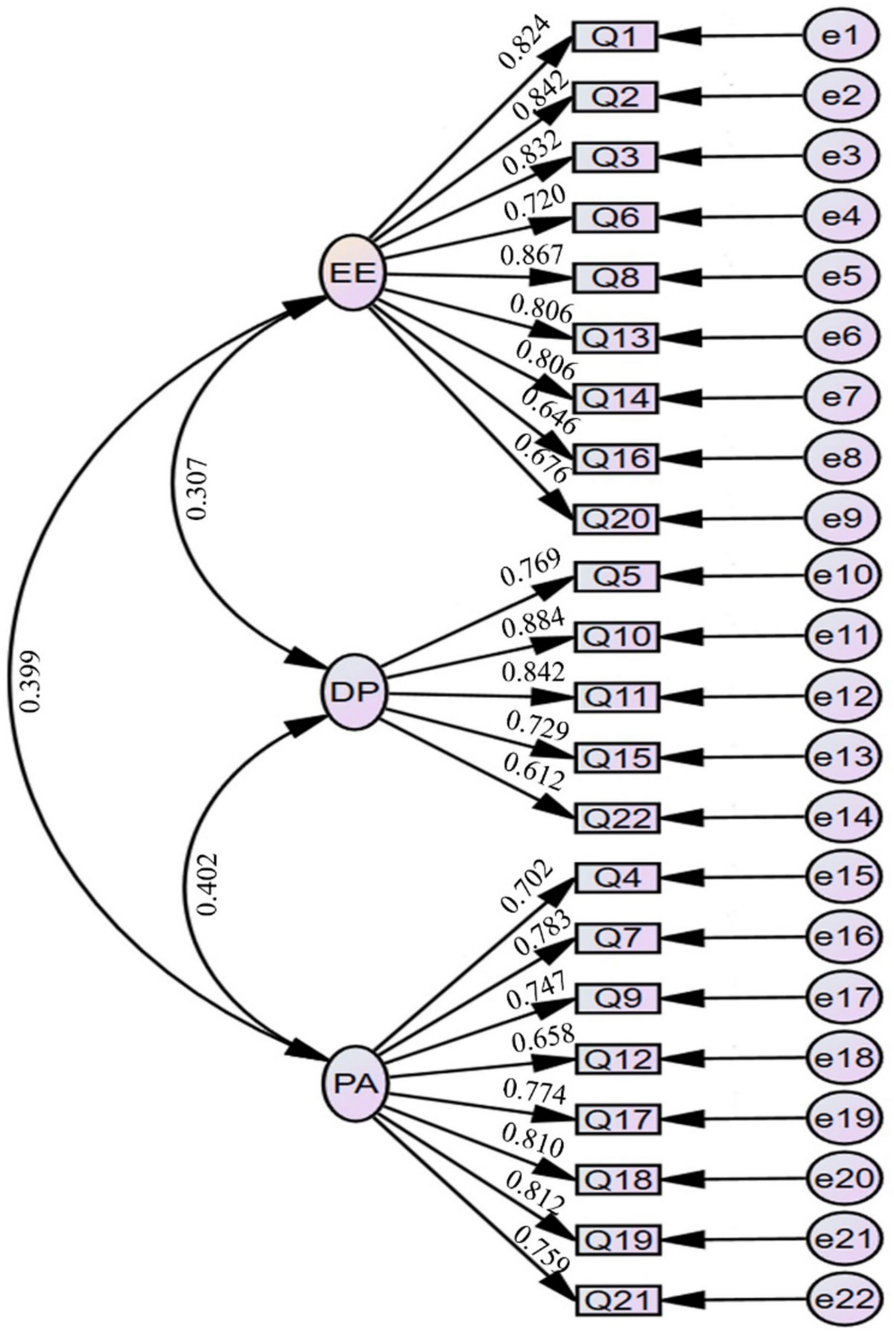
The plot of the confirmatory factor analysis model for MBI-HSS. EE, emotional exhaustion; DP, depersonalization; PA, personal accomplishment; MBI-HSS, Maslach Burnout Inventory-Human Services Survey.

To explore the validity of MBI-HSS, we also conducted a CFA on it, as shown in Figure 1. Due to the three sections comprising MBI-HSS, we conducted separate analyses for each part. The results showed that the fit indices for EE, and DP were within an acceptable range, indicating that they all had a good one-factor structure. Additionally, we also performed CFA on multiple structural models of MBI-HSS and found that both the two-factor and three-factor models exhibited acceptable fit indices, with the three-factor model showing the best fit. This further validates the structural validity of MBI-HSS (Table 3).

**Table 3:** Fitting index calculated by CFA.

Items	χ^2^	df	χ^2^/df	*P*	RMSEA	CFI	TLI
EE	531.241	271	1.970	<0.001	0.058	0.805	0.840
DP	1036.564	427	2.428	<0.001	0.095	0.804	0.815
PA	1264.777	352	3.593	<0.001	0.103	0.728	0.755
MBI-HSS							
Single factor model	792.988	209	3.794	<0.001	0.159	0.729	0.689
Two factor model	601.012	208	2.889	<0.001	0.074	0.862	0.825
Three factor model	397.081	206	1.928	<0.001	0.048	0.914	0.935

Single factor model: EE + PA + DP; Two factor model 1: EE + PA, DP; Three factor model: EE, PA, DP. df, Degrees of freedom; CFI, comparative fit index; TLI, Tucker-Lewis index; RMSEA, root mean square error of approximation; EE, emotional exhaustion; DP, depersonalization; PA, personal accomplishment; MBI-HSS, Maslach Burnout Inventory-Human Services Survey.

Due to the good construct validity of MBI-HSS in this study, we examined its convergent validity and combined reliability. We also calculated AVE and CR based on SFL. The results showed that the AVE for EE, DP, and PA were 0.614, 0.598, and 0.573, while their CR values were 0.934, 0.880, and 0.915, all falling within an acceptable range. These findings demonstrate the excellent convergence validity and composite reliability of MBI-HSS (Table 4).

**Table 4:** The convergence validity and composite reliability of MBI-HSS.

Path information			SFL	AVE	CR
EE				0.614	0.934
Q1	<---	EE	0.824		
Q2	<---	EE	0.842		
Q3	<---	EE	0.832		
Q6	<---	EE	0.720		
Q8	<---	EE	0.867		
Q13	<---	EE	0.806		
Q14	<---	EE	0.806		
Q16	<---	EE	0.646		
Q20	<---	EE	0.676		
DP				0.598	0.880
Q5	<---	DP	0.769		
Q10	<---	DP	0.884		
Q11	<---	DP	0.842		
Q15	<---	DP	0.729		
Q55	<---	DP	0.612		
PA				0.573	0.915
Q4	<---	PA	0.702		
Q7	<---	PA	0.783		
Q9	<---	PA	0.747		
Q12	<---	PA	0.658		
Q17	<---	PA	0.774		
Q18	<---	PA	0.810		
Q19	<---	PA	0.812		
Q21	<---	PA	0.759		

SFL, Standardized Factor Loadings; AVE, Average Variance Extracted; CR, Composite Reliability; EE, emotional exhaustion; DP, depersonalization; PA, personal accomplishment; MBI-HSS, Maslach Burnout Inventory-Human Services Survey.

Finally, we analyzed the discriminant validity of MBI-HSS. During the examination, we found that the correlation coefficients between any two factors were all smaller than the square root of AVE values of EE, DP, and PA, indicating that there is good discriminant validity between any two factors (Table 5).

**Table 5:** The discriminant validity test of MBI-HSS.

	EE	DP	PA
EE	**0.614**		
DP	0.307	**0.598**	
PA	0.399	0.402	**0.573**
AVE^2^	0.784	0.773	0.757

EE, emotional exhaustion; DP, depersonalization; PA, personal accomplishment; MBI-HSS, Maslach Burnout Inventory-Human Services Survey.

The bold values represent the AVE of EE, DP, and PA.

### 3.4. Burnout, anxiety, and depression levels

The K-S test confirmed that all continuous variables in this study followed a Gaussian distribution. The mean scores for EE, DP, PA, GAD-7, and PHQ-9 among all participants were 28.15 (SD = 11.45), 111.28 (SD = 5.47), 37.49 (SD = 10.03), 5.59 (SD = 2.50), and 7.48 (SD = 2.20) respectively. Moreover, the scores of doctors in terms of EE, DP, GAD-7, and PHQ-9 were all significantly higher than those of nurses (all *p* < 0.05).

The rates of EE, DP, and diminished PA among all participants were 52.4, 55.3, and 30.6%, respectively. When analyzed separately, doctors exhibited higher prevalence rates of EE, DP, and diminished PA at 61.1, 61.8, and 29.5%, respectively, while nurses had rates of 44.1, 49.1, and 31.7%, respectively. When employing the relatively lenient approach, a total of 987 participants (65.6%) were found to experience burnout. Among them, 530 (72.5%) were doctors, and 457 (59.0%) were nurses. The burnout rate among doctors was significantly higher than that of nurses (*p* < 0.001). Even when using the most stringent diagnostic criteria, there were still 270 medical staff (17.9%) experiencing occupational burnout, including 149 doctors (20.4%) and 121 nurses (15.6%). Doctors also have a higher rate of burnout (*p* < 0.001).

In this study, a total of 200 (13.3%) medical staff were found to experience anxiety, including 98 (13.4%) doctors and 102 (13.2%) nurses. Additionally, 388 participants (25.8%) reported experiencing depression, comprising 199 (27.2%) doctors and 189 (24.4%) nurses. There was no significant difference in anxiety (*p* = 0.896) and depression (*p* = 0.214) among medical staff of different occupations (Table 6).

**Table 6:** The level of burnout, anxiety, and depression.

Items, *n* (%)	Occupation			*p* value
	Total	Doctor	Nurse	
	*n* = 1,505	*n* = 731	*n* = 774	
EE score, mean (SD)	28.15 (11.45)	30.11 (11.06)	26.30 (11.50)	<0.001
DP score, mean (SD)	11.28 (5.47)	11.90 (5.37)	10.68 (5.51)	<0.001
PA score, mean (SD)	37.49 (10.03)	37.32 (9.39)	37.64 (10.60)	0.543
GAD-7 score, mean (SD)	5.59 (2.50)	5.95 (2.44)	5.24 (2.52)	0.002
PHQ-9 score, mean (SD)	7.48 (2.20)	7.87 (2.03)	7.11 (2.34)	0.005
EE				<0.001
Yes	788 (52.4)	447 (61.1)	341 (44.1)	
No	717 (47.6)	284 (38.9)	433 (55.9)	
DP				<0.001
Yes	832 (55.3)	452 (61.8)	380 (49.1)	
No	673 (44.7)	279 (38.2)	394 (50.9)	
Diminished PA				0.376
Yes	461 (30.6)	216 (29.5)	245 (31.7)	
No	1,044 (69.4)	515 (70.5)	529 (68.3)	
Burnout^1#^				<0.001
Yes	987 (65.6)	530 (72.5)	457 (59.0)	
No	518 (34.4)	201 (27.5)	317 (41.0)	
Burnout^2#^				0.016
Yes	270 (17.9)	149 (20.4)	121 (15.6)	
No	1,235 (82.1)	582 (79.6)	653 (84.4)	
Anxiety				0.896
Yes	200 (13.3)	98 (13.4)	102 (13.2)	
No	1,305 (86.7)	633 (86.6)	672 (86.8)	
Depression				0.214
Yes	388 (25.8)	199 (27.2)	189 (24.4)	
No	74.2 (1505)	532 (72.8)	585 (75.6)	

#Burnout^1^: The participants with EE score ≥ 27 or DP score ≥ 10. Burnout^2^: The participants with EE score ≥ 27, DP score ≥ 10, and PA score < 33. SD, standard deviation; EE, emotional exhaustion; DP, depersonalization; PA, personal accomplishment (PA); GAD-7, Generalized Anxiety Disorder Questionnaire 7; PHQ-9, Patient Health Questionnaire 9; MBI-HSS-MP, Maslach Burnout Inventory-Human Services Survey for Medical Personnel.

After analyzing the data, we found significant differences between doctors and nurses not only in baseline characteristics but also in their scores for burnout, anxiety, and depression, as well as the prevalence of these conditions. Particularly, there were significant differences between the two groups in terms of gender (*p* < 0.001) and educational level (*p* < 0.001), which were considered important underlying factors influencing psychological status. Therefore, to reduce potential bias in the results, we conducted separate analyses for doctors and nurses in all subsequent analyses. In addition, to explore burnout more accurately, we used the more stringent diagnostic criteria in all subsequent analyses.

### 3.5. The univariate and multivariate regression analysis

To investigate the factors contributing to occupational burnout, we conducted logistic regression analysis on the psychological factors of medical personnel. In doctors, we observed significant associations between occupational burnout and several factors, including age, marriage, relationship with family members, obtaining psychological support, personal and family members’ health conditions, exceeding legal working hours, promotion pressure, and insufficient sleep (all *p* < 0.05). To prevent result bias, we calculated the tolerance and VIF of all burnouts related factors before multifactor analysis and found that there was no multicollinearity between them (all tolerance >0.1 and all VIF <10) (Table 7). After incorporating these factors into multivariate analysis, we found that lower age (HR = 1.046, *p* = 0.003), not obtaining psychological support (HR = 1.462, *p* = 0.042), having medium or poor health conditions (HR = 1.843, *p* = 0.015), relationship with family members (HR = 1.911, *p* = 0.033), no income increase (HR = 2.064, *p* = 0.021), promotion pressure (HR = 1.793, *p* = 0.036), and experiencing insufficient sleep (HR = 2.176, *p* = 0.022) were identified as significant risk factors for occupational burnout (Table 8).

**Table 7:** The tolerance and VIF of all burnout related factors.

Items	Doctor		Nurse	
	Tolerance	VIF	Tolerance	VIF
Age	0.606	1.651		
Marriage	0.628	1.591		
Relationship with family members	0.924	1.083	0.921	1.086
Obtaining psychological support	0.913	1.095	0.934	1.071
Health condition	0.629	1.590	0.658	1.520
Health condition of family members	0.658	1.520	0.666	1.503
Income increase	0.942	1.061	0.939	1.064
Exceeding legal working hours	0.879	1.138	0.908	1.101
Promotion pressure	0.762	1.312	0.888	1.126
Insufficient sleep	0.770	1.299	0.857	1.167

VIF, Variance Inflation Factor.

**Table 8:** Univariate and multivariate analysis of burnout among doctors.

Items	Doctor			
	Univariate analysis		Multivariate analysis	
	HR (95% CI)	*P*	HR (95% CI)	*P*
Age	1.049 (1.026–1.073)	<0.001	1.046 (1.015–1.077)	0.003
Sex				
Male	Ref			
Female	1.072 (0.748–1.536)	0.717		
Department				
Non-Surgery	Ref			
Surgery	1.207 (0.842–1.732)	0.306		
Educational level				
Undergraduate or below	Ref			
Master or above	1.133 (0.785–1.635)	0.506		
Marriage				
Yes	Ref		Ref	
No	1.709 (1.160–2.516)	0.007	1.148 (0.689–1.915)	0.596
Relationship with family members				
Well	Ref		Ref	
Medium + Poor	2.749 (1.591–4.749)	<0.001	1.911 (1.054–3.467)	0.033
Obtaining psychological support				
Yes	Ref		Ref	
No	1.569 (1.006–2.447)	0.047	1.462 (1.116–2.286)	0.042
Health condition				
Well	Ref		Ref	
Medium + Poor	2.587 (1.775–3.771)	<0.001	1.843 (1.128–3.012)	0.015
Health condition of family members				
Well	Ref		Ref	
Medium + Poor	2.039 (1.831–3.011)	<0.001	1.091 (0.658–1.808)	0.737
Participate in epidemic prevention				
No	Ref			
Yes	1.230 (0.822–1.840)	0.314		
Income increase				
Yes	Ref		Ref	
No	2.557 (1.424–4.592)	0.002	2.064 (1.113–3.829)	0.021
Exceeding legal working hours				
No	Ref		Ref	
Yes	1.772 (1.174–2.674)	0.006	1.243 (0.791–1.951)	0.346
Promotion pressure				
No	Ref		Ref	
Yes	2.820 (1.737–4.578)	<0.001	1.793 (1.038–3.095)	0.036
Insufficient sleep				
No	Ref		Ref	
Yes	3.503 (1.924–6.377)	<0.001	2.176 (1.117–4.241)	0.022

HR, Hazard Ratios; CI, Confidence Intervals.

For nurses, the following factors were found to be associated with burnout: relationship with family members, obtaining psychological support, personal or family members’ health conditions, income changes, exceeding legal working hours, promotion pressure, and insufficient sleep (All *p* < 0.05). At the same time, these factors also did not exhibit multicollinearity (all tolerance >0.1 and all VIF <10) (Table 7). Furthermore, we found that medium or poor relationship with family members (HR = 2.395, *p* = 0.007), obtaining psychological support (HR = 1.606, *p* = 0.025), medium or poor health condition (HR = 2.013, *p* = 0.008), no income increase (HR = 2.021, *p* = 0.008), exceeding legal working hours (HR = 1.686, *p* = 0.016), and insufficient sleep (HR = 2.863, *p* = 0.003) were independent factors influencing burnout (Table 9).

**Table 9:** Univariate and multivariate analysis of burnout among nurses.

Items	Nurse			
	Univariate analysis		Multivariate analysis	
	HR (95% CI)	*P*	HR (95% CI)	*P*
Age	1.030 (0.999–1.062)	0.055		
Department				
Non-Surgery	Ref			
Surgery	1.263 (0.843–1.891)	0.257		
Marriage				
Yes	Ref			
No	1.125 (0.699–1.813)	0.627		
Relationship with family members				
Well	Ref		Ref	
Medium + Poor	3.496 (1.966–6.217)	<0.001	2.395 (1.263–4.541)	0.007
Obtaining psychological support				
Yes	Ref		Ref	
No	1.845 (1.201–2.834)	0.005	1.606 (1.125–2.367)	0.025
Health condition				
Well	Ref		Ref	
Medium + Poor	2.953 (1.971–4.424)	<0.001	2.013 (1.205–3.365)	0.008
Health condition of family members				
Well	Ref		Ref	
Medium + Poor	2.171 (1.416–3.328)	<0.001	1.008 (0.580–1.752)	0.977
Participate in epidemic prevention				
No	Ref			
Yes	1.230 (0.782–1.936)	0.370		
Income increase				
Yes	Ref		Ref	
No	2.271 (1.392–3.705)	0.001	2.021 (1.202–3.398)	0.008
Exceeding legal working hours				
No	Ref		Ref	
Yes	2.344 (1.581–3.477)	<0.001	1.686 (1.102–2.579)	0.016
Promotion pressure				
No	Ref		Ref	
Yes	1.965 (1.296–2.980)	0.001	1.308 (0.833–2.053)	0.243
Insufficient sleep				
No	Ref		Ref	
Yes	4.692 (2.404–9.155)	<0.001	2.863 (1.417–5.782)	0.003

HR, Hazard Ratios; CI, Confidence Intervals.

### 3.6. The nomograms predicting the probability of burnout

**Figure 2:**
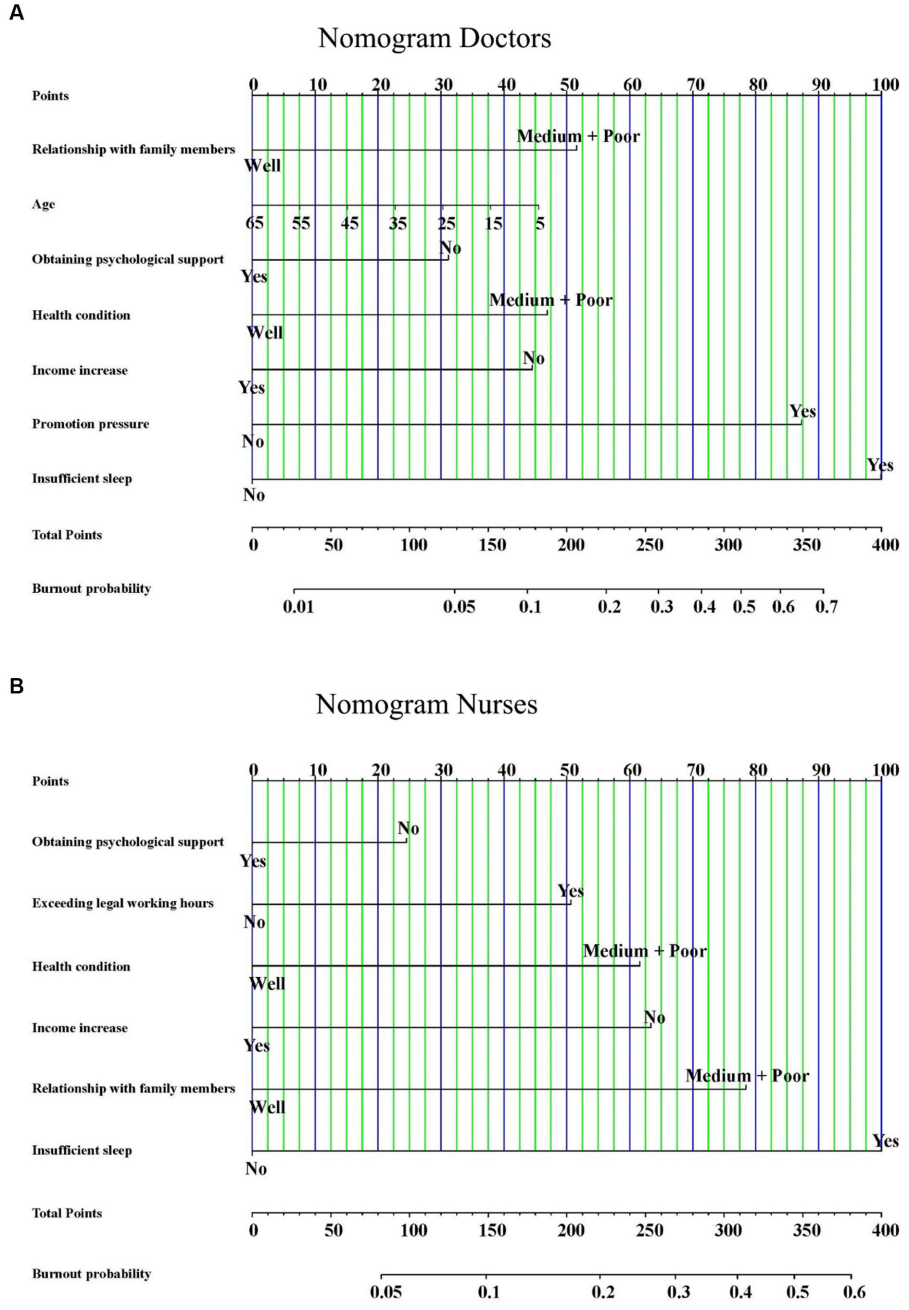
The nomograms for predicting burnout of doctors **(A)** and nurses **(B)**.

**Figure 3:**
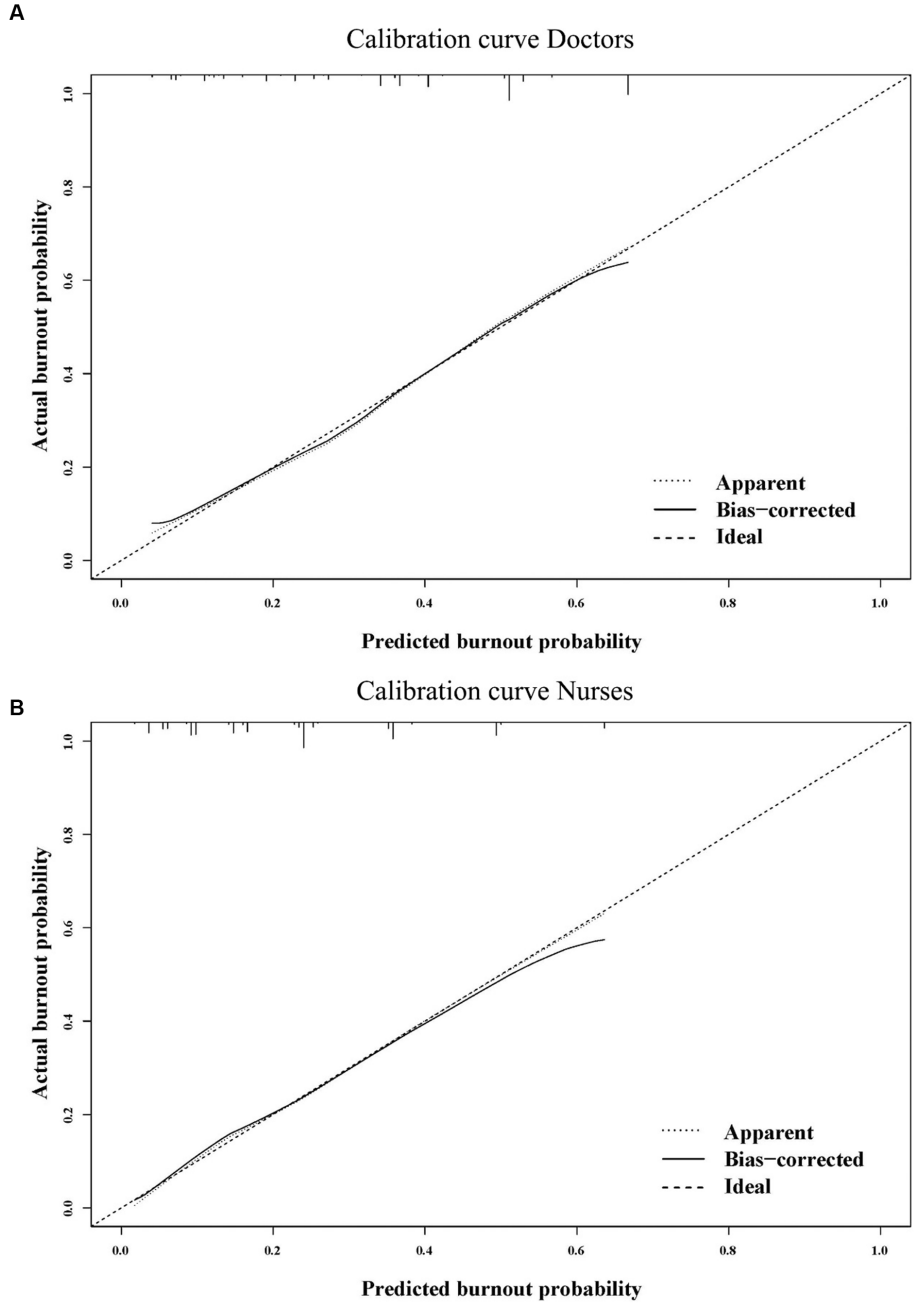
The calibration curves for nomograms of doctors **(A)** and nurses **(B)**.

We constructed nomograms to predict the probability of occupational burnout for doctors and nurses based on the independent influencing factors (Figures 2A,B). Furthermore, we also conducted bootstrap correction for the nomograms and plotted calibration curves. The C-index for the nomograms was 0.832 and 0.843, respectively. Meanwhile, the calibration curves demonstrated good consistency between predicted and actual probabilities (Figures 3A,B).

### 3.7. The relationship between burnout, anxiety, and depression

**Figure 4:**
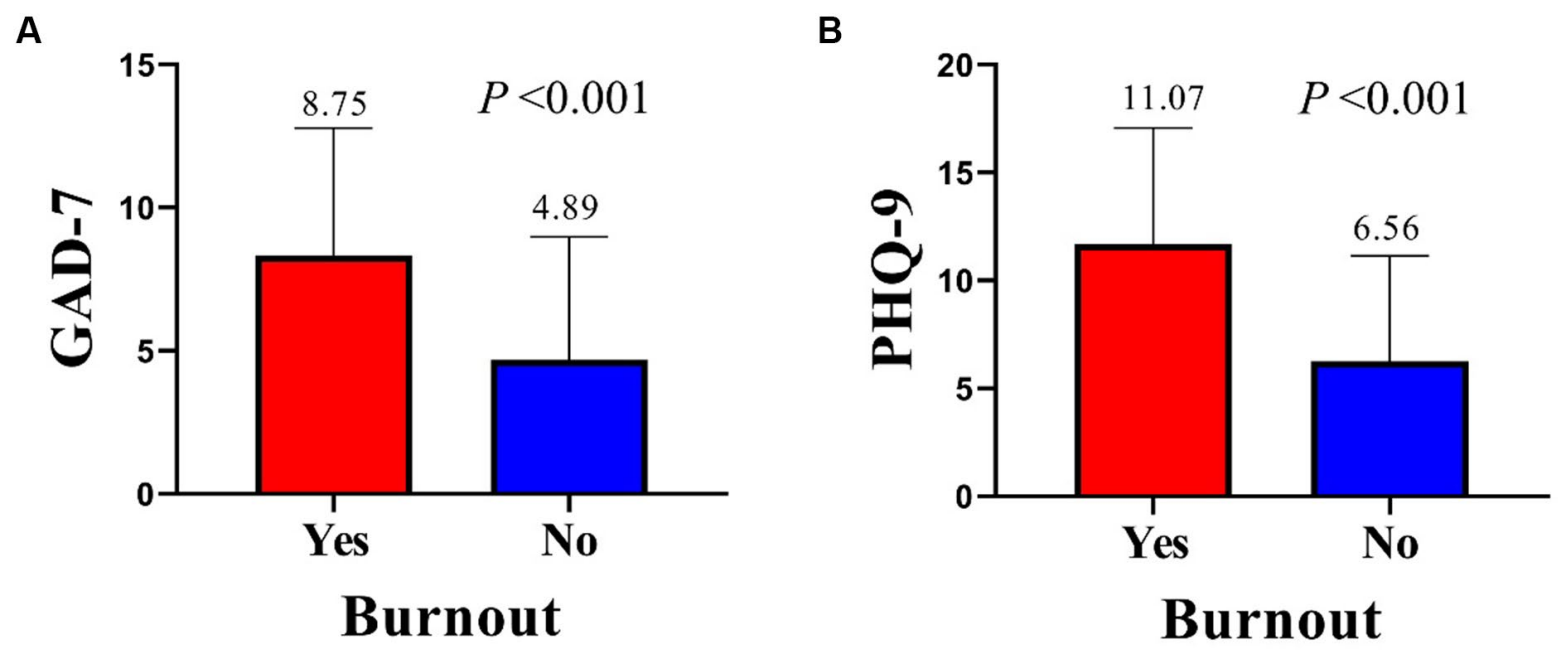
Differences in GAD-7 **(A)** and PHQ-9 **(B)** score. GAD-7, Generalized Anxiety Disorder Questionnaire 7; PHQ-9, Patient Health Questionnaire 9.

We further explored the relationship between burnout and anxiety and depression. Medical staff with burnout had significantly higher average scores on the GAD-7 (8.75 vs. 4.89, *p* < 0.001) and PHQ-9 (11.07 vs. 6.56, *p* < 0.001) compared to those without occupational burnout (Figures 4A,B).

**Figure 5:**
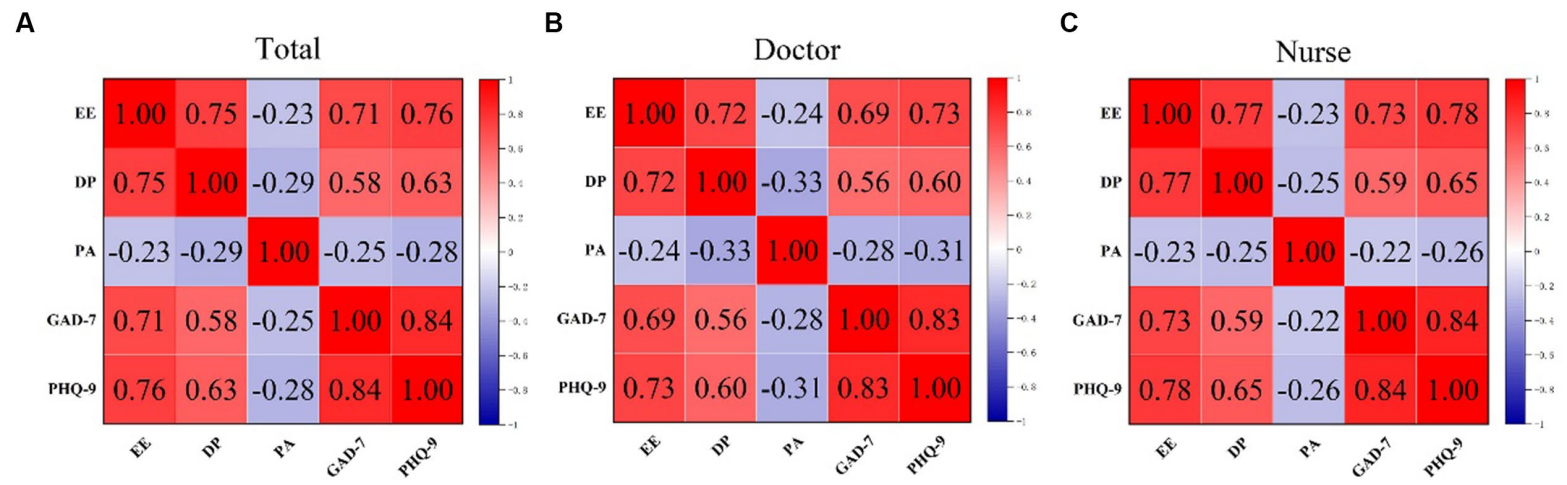
The Pearson analyses for the total sample **(A)**, doctors **(B)**, and nurses **(C)**. EE, emotional exhaustion; DP, depersonalization; PA, personal accomplishment (PA); GAD-7, Generalized Anxiety Disorder Questionnaire 7; PHQ-9, Patient Health Questionnaire 9.

In addition, as the score of EE, DP, PA, GAD-7, and PHQ-9 all followed a Gaussian distribution, we explored their relationships in the context of continuous variables. Pearson correlation analysis revealed that in both the total sample and among doctors or nurses separately, there were significant positive correlations between any two factors among the score of PA, DP, GAD-7, and PHQ-9 (all R > 0.3, *p* < 0.001). Furthermore, the score of PA also decreased with an increase in any of the other factors (*p* < 0.05) (Figures 5A–C). This means that there was a significant linear correlation between burnout and anxiety and depression.

Finally, we analyzed the anxiety and depression status among medical personnel with burnout. The results indicated that burnout was significantly positively correlated not only with the presence of anxiety and depression symptoms but also with the severity of anxiety and depression as classified (All *p* < 0.001) (Table 10).

**Table 10:** The correlation analysis between occupational burnout and anxiety and depression.

Items, *n* (%)	Total			Doctor			Nurse		
	Burnout			Burnout			Burnout		
	Yes	No	*P*	Yes	No	*P*	Yes	No	*P*
Anxiety level			<0.001			<0.001			<0.001
Normal	25 (9.3)	296 (24.0)		9 (6.0)	137 (23.5)		16 (13.2)	159 (24.3)	
Mild	161 (59.6)	823 (66.6)		93 (62.4)	394 (67.7)		68 (56.2)	429 (65.7)	
Moderate	59 (21.9)	90 (7.3)		30 (20.1)	39 (6.7)		29 (24.0)	51 (7.8)	
Severe	25 (9.3)	26 (2.1)		17 (11.4)	12 (2.1)		8 (6.6)	14 (2.1)	
Anxiety disorder			<0.001			<0.001			<0.001
Yes	84 (31.1)	116 (9.4)		47 (31.5)	51 (8.8)		37 (30.6)	65 (10.0)	
No	186 (68.9)	1,119 (90.6)		102 (68.5)	531 (91.2)		84 (69.4)	588 (90.0)	
Depression level			<0.001			<0.001			<0.001
Normal	8 (3.0)	427 (34.6)		3 (2.0)	167 (28.7)		5 (4.1)	260 (39.8)	
Mild	122 (45.2)	560 (45.3)		71 (47.7)	291 (50.0)		51 (42.1)	269 (41.2)	
Moderate	84 (31.1)	183 (14.8)		45 (30.2)	96 (16.5)		39 (13.3)	87 (13.3)	
Severe	56 (20.7)	65 (5.3)		30 (20.1)	28 (4.8)		26 (21.5)	37 (5.7)	
Depression disorder			<0.001			<0.001			<0.001
Yes	140 (51.9)	248 (20.1)		75 (50.3)	124 (21.3)		65 (53.7)	124 (19.0)	
No	130 (48.1)	987 (79.9)		74 (49.7)	458 (78.7)		56 (46.3)	529 (81.0)	

## 4. Discussion

After the end of the COVID-19 pandemic, medical staff immediately confronted a substantial increase in their workload, with prolonged emotional exhaustion exacerbating their level of burnout, significantly impacting their health. Therefore, it is crucial to investigate the burnout status and its associated factors among medical staff during this period. In addition, previous studies have preliminarily revealed a certain degree of connection between burnout, anxiety, and depression (31–33). Considering the potentially severe consequences of anxiety and depression, it is equally important to explore the anxiety and depression levels of medical staff during this period and their relationship with burnout.

There are various methods for assessing burnout among medical staff, with various versions of MBI being the most used (34, 35). Bassam and his colleagues conducted a cross-sectional survey to explore the adaptability of the Arabic version of MBI-HSS among dentists. They obtained acceptable Cronbach’s α, demonstrating good reliability of the MBI-HSS. Additionally, they conducted exploratory factor analysis (EFA) and CFA for MBI-HSS and found that the Arabic version of MBI-HSS performed well in the study (36). Other studies exploring the reliability of MBI-HSS have also shown its good adaptability (37, 38). To ensure the effectiveness of all subsequent analyses, we first explored the reliability of the Chinese version of MBI-HSS among healthcare workers. The Cronbach’s α for EE, DP, PA, and the overall MBI-HSS were 0.932, 0.818, 0.877, and 0.830, respectively, indicating their high internal consistency reliability. In addition, all relevant indices obtained from CFA were within an acceptable or excellent range, indicating its good structural validity, convergent validity, and discriminant validity. This evidence collectively demonstrated the excellent performance of the Chinese version of MBI-HSS among medical staff.

At present, there is still no universally recognized burnout diagnostic criterion based on MBI-HSS. As a result, the rates of burnout calculated by different studies have significant heterogeneity (30). Researchers commonly report the levels and prevalence rates of EE, DP, and diminished PA separately (39). In 2021, Tang and his colleagues conducted a survey using MBI-HSS to assess the psychological state of healthcare workers in Shanghai during the COVID-19 pandemic. They found that the medical staff’s occupational burnout was significantly higher compared to the pre-pandemic levels. The average scores for EE, DP, and PA were 23.09 ± 9.24, 7.97 ± 4.82, and 24.74 ± 6.62, respectively (40). Lasalvia et al. conducted a cross-sectional survey among healthcare workers in Northeast Italy using MBI-GS. The prevalence rates of emotional exhaustion, professional efficacy, and cynicism in MBI-GS were 38.3, 46.5, and 26.5%, respectively, indicating relatively high levels of burnout among the participants (41). Houdmont et al. conducted a survey on burnout among British surgeons, and their findings were similar. Their cross-sectional study, using MBI-HSS-MP, revealed prevalence rates of 56.9% for EE, 48.5% for DP, and 14.3% for reduced PA, indicating similarly high levels of burnout among the participants (42). During COVID-19, medical staff, as the primary force in the fight against the pandemic, faced immense pressure. The high risk of infection, heavy workloads, shortages of medical resources, and personal protective equipment (PPE) that could potentially harm healthcare workers all contributed to elevated levels of occupational burnout among them (43, 44). Many other studies have shown that this situation was widespread worldwide (45–47). In this study, we found significantly higher average score and prevalence rate of EE, DP, and reduced PA at 28.15 ± 11.45, 11.28 ± 5.47, and 26.30 ± 11.50, respectively, with prevalence rates of 52.4, 55.3, and 30.6%. This indicates that the levels of burnout among medical staff were comparable or even higher during the COVID-19 period. The possible reasons for this could be that medical staff have recently experienced immense pressure during the COVID-19 prevention and control efforts, leading to the accumulation of various negative emotions. The immediate onset of heavy workloads has significantly exacerbated the psychological issues among healthcare workers, resulting in severe occupational burnout.

Despite significant heterogeneity in the results of burnout status determined by MBI-HSS, to explore the related factors of burnout more conveniently, we still diagnosed burnout using two commonly used standards. The prevalence rates of burnout obtained from the relatively lenient criterion and the relatively strict criterion were 65.6 and 17.9%, respectively, having a substantial difference. To ensure the accuracy of the results, we conducted all subsequent analyses using the more stringent criterion. The multivariate analysis showed that obtaining psychological support, health condition, relationship with family members, and insufficient sleep were influencing factors for burnout among both doctors and nurses. Additionally, age and promotion pressure were also associated with burnout among doctors, while exceeding legal working hours was related to burnout among nurses.

During the COVID-19 period, the significant deterioration of emotions among medical staff prompted hospitals and related organizations to actively provide various forms of psychological support, leading to significant positive outcomes (48). Ding et al. conducted in-depth interviews with 15 nurses working on the frontline during COVID-19 and found that providing appropriate psychological care could improve their mental health (49). The findings of this study suggested that even after the end of COVID-19, healthcare workers still required a certain level of psychological care to alleviate their occupational burnout or other negative emotions. On the other hand, following the lifting of COVID-19 lockdown measures, many medical staff have been infected with COVID-19. However, due to the immense workload, they often cannot get sufficient time for recovery before returning to the hospital, leading to prolonged exhaustion. Furthermore, a substantial body of previous research has also confirmed that health condition was a significant contributing factor to various emotional disorders (50–54). Therefore, health condition was also a crucial factor contributing to burnout among healthcare workers during this period. Family support was an effective means of alleviating various emotional disorders, including burnout, anxiety, and depression (55, 56). Tang and his colleagues conducted a cross-sectional survey of 8,135 primary healthcare workers and found that family support was a significant protective factor against burnout (57). The results of this study were consistent with this finding, as poorer family relationships resulted in medical staff receiving less family support, making them more susceptible to burnout. Insufficient sleep has consistently been identified as a significant contributing factor to various psychological issues, especially burnout (58). Stewart and Kancherla’s reviews specifically discussed the role of sleep status in physician burnout and provided ample evidence to demonstrate its significant promotion of burnout (59, 60). Similarly, in this study, insufficient sleep was also identified as the most significant factor leading to burnout. Doctors often faced higher levels of promotion pressure. However, the heavy workload prevented them from investing more energy, leading to the development of anxiety. Additionally, young doctors have more responsibilities at work and heavier family burdens. These all contributed to the accumulation of negative emotions. Therefore, age and promotion pressure were also factors leading to burnout among doctors. Exceeding legal working hours not only exhausted nurses but also speeded feelings of dissatisfaction and contributed to nurse burnout.

The relationship between burnout and anxiety-depression was complex. Some perspectives suggested that there was a certain degree of overlap between burnout and anxiety-depression (61). However, a study conducted by Koutsimani et al. in 2019, after collecting and analyzing all relevant literature from 2007 to 2018, found no significant overlap between burnout and depression, as well as between burnout and anxiety (62). This created the conditions for further research into their relationship. In this study, the analysis of burnout, anxiety, and depression also revealed a significant linear correlation between them. The exacerbation of burnout might lead to the occurrence of anxiety and depression. One possible reason was the presence of overlapping factors that have been identified in previous research as contributing to anxiety, depression, and burnout (63–66). Additionally, anxiety and depression were the result of long-term accumulation of negative emotions, and as burnout was a severe negative emotion, it naturally leaded to the development of anxiety and depression. Due to the potential serious consequences of anxiety and depression, timely intervention for burnout becomes even more crucial (67–69).

Given the potential for severe consequences, including suicide, resulting from severe anxiety and depression, continued attention to the burnout of medical staff has become equally important (70). During COVID-19, various organizations have had well-established psychological support strategies for medical staff, and emerging remote psychological support methods not only significantly improve efficiency but also yield good results (71, 72). These strategies remain necessary after the end of COVID-19.

This study still had some unavoidable limitations. Firstly, although 1,505 participants were included, it is still a relatively small sample compared to the total number of medical staff. This might result in limited representativeness and potential biases in the study’s findings. Secondly, there was still a lack of universally recognized methods for detecting and diagnosing occupational burnout, leading to significant variations in results among different studies. Lastly, the psychological-related questions included in the survey might not cover all factors that contribute to burnout. The conclusions of this study need further validation in larger samples and more well-designed studies.

## 5. Conclusion

After the end of COVID-19, medical staff in high workload environments were facing severe burnout, which might lead to anxiety and depression. The occupational burnout of medical staff needed to be taken seriously and actively intervened.

## Data availability statement

The raw data supporting the conclusions of this article will be made available by the authors, without undue reservation.

## Ethics statement

The studies involving humans were approved by the Ethics Committee of Cancer Hospital of Harbin Medical University. The studies were conducted in accordance with the local legislation and institutional requirements. The ethics committee/institutional review board waived the requirement of written informed consent for participation from the participants or the participants’ legal guardians/next of kin because this was an anonymous cross-sectional survey that does not require informed consent.

## Author contributions

HaS: Writing – original draft, Writing – review & editing. TZ: Writing – original draft, Writing – review & editing. XW: Data curation, Investigation, Writing – review & editing. CW: Data curation, Investigation, Writing – review & editing. MZ: Methodology, Supervision, Writing – review & editing. HoS: Funding acquisition, Project administration, Resources, Writing – review & editing.

## Footnotes

^1^Supported by www.wjx.cn.

^2^https://www.ibm.com, accessed on June 12, 2023.

^3^https://www.r-project.org, accessed on June 14, 2023.

^4^http://www.statmodel.com, accessed on June 13, 2023.

^5^https://www.ibm.com, accessed on June 13, 2023.
