# Supplementary material for: The occupational burnout among medical staff with high workloads after the COVID-19 and its association with anxiety and depression
Source: Front Public Health. 2023 Oct 26;11:1270634. doi: 10.3389/fpubh.2023.1270634 (PMC10639132; doi:10.3389/fpubh.2023.1270634)
Supplement: Supplementary file 1 [file Data_Sheet_1.docx]

**S1** Chinese version of Maslach Burnout Inventory-Human Services Survey

1. 工作有时使我情绪低落

0从未有过

1极少数时候(一年中有几次或更少)

2少数时候（一个月一次或更少）

3稍多时候（一个月中有几次）

4多数时候（一个星期一次）

5几乎每天（一个星期中有几次）

6每天

2. 工作一天，我感到非常疲惫

0从未有过

1极少数时候(一年中有几次或更少)

2少数时候（一个月一次或更少）

3稍多时候（一个月中有几次）

4多数时候（一个星期一次）

5几乎每天（一个星期中有几次）

6每天

3. 早上起床，感到很疲乏，但仍要面对当天的工作

0从未有过

1极少数时候(一年中有几次或更少)

2少数时候（一个月一次或更少）

3稍多时候（一个月中有几次）

4多数时候（一个星期一次）

5几乎每天（一个星期中有几次）

6每天

4. 我能轻易的明白病人对事物的感受

0从未有过

1极少数时候(一年中有几次或更少)

2少数时候（一个月一次或更少）

3稍多时候（一个月中有几次）

4多数时候（一个星期一次）

5几乎每天（一个星期中有几次）

6每天

5. 有时把病人、同事当物品一样看待，而不是面对一个人

0从未有过

1极少数时候(一年中有几次或更少)

2少数时候（一个月一次或更少）

3稍多时候（一个月中有几次）

4多数时候（一个星期一次）

5几乎每天（一个星期中有几次）

6每天

6. 整体和人打交道的工作，对我来说是一种负担

0从未有过

1极少数时候(一年中有几次或更少)

2少数时候（一个月一次或更少）

3稍多时候（一个月中有几次）

4多数时候（一个星期一次）

5几乎每天（一个星期中有几次）

6每天

7. 我能有效的处理病人、同事的问题

0从未有过

1极少数时候(一年中有几次或更少)

2少数时候（一个月一次或更少）

3稍多时候（一个月中有几次）

4多数时候（一个星期一次）

5几乎每天（一个星期中有几次）

6每天

8. 工作使我耗尽了心力

0从未有过

1极少数时候(一年中有几次或更少)

2少数时候（一个月一次或更少）

3稍多时候（一个月中有几次）

4多数时候（一个星期一次）

5几乎每天（一个星期中有几次）

6每天

9. 我觉得自己的工作对他人的生活发挥了积极的作用

0从未有过

1极少数时候(一年中有几次或更少)

2少数时候（一个月一次或更少）

3稍多时候（一个月中有几次）

4多数时候（一个星期一次）

5几乎每天（一个星期中有几次）

6每天

10. 自从从事这份工作后,我对人越来越冷漠了

0从未有过

1极少数时候(一年中有几次或更少)

2少数时候（一个月一次或更少）

3稍多时候（一个月中有几次）

4多数时候（一个星期一次）

5几乎每天（一个星期中有几次）

6每天

11. 我担心这份工作会使我变成一个硬心肠的人

0从未有过

1极少数时候(一年中有几次或更少)

2少数时候（一个月一次或更少）

3稍多时候（一个月中有几次）

4多数时候（一个星期一次）

5几乎每天（一个星期中有几次）

6每天

12. 我觉得自己精力充沛

0从未有过

1极少数时候(一年中有几次或更少)

2少数时候（一个月一次或更少）

3稍多时候（一个月中有几次）

4多数时候（一个星期一次）

5几乎每天（一个星期中有几次）

6每天

13. 我感到我在工作中受到挫折

0从未有过

1极少数时候(一年中有几次或更少)

2少数时候（一个月一次或更少）

3稍多时候（一个月中有几次）

4多数时候（一个星期一次）

5几乎每天（一个星期中有几次）

6每天

14. 我感到自己的工作过于辛苦

0从未有过

1极少数时候(一年中有几次或更少)

2少数时候（一个月一次或更少）

3稍多时候（一个月中有几次）

4多数时候（一个星期一次）

5几乎每天（一个星期中有几次）

6每天

15. 我不太重视病人提出的需求

0从未有过

1极少数时候(一年中有几次或更少)

2少数时候（一个月一次或更少）

3稍多时候（一个月中有几次）

4多数时候（一个星期一次）

5几乎每天（一个星期中有几次）

6每天

16. 与其他人一起工作使我感到有很大的压力

0从未有过

1极少数时候(一年中有几次或更少)

2少数时候（一个月一次或更少）

3稍多时候（一个月中有几次）

4多数时候（一个星期一次）

5几乎每天（一个星期中有几次）

6每天

17. 与病人一起时我可以轻易地营造轻松的气氛

0从未有过

1极少数时候(一年中有几次或更少)

2少数时候（一个月一次或更少）

3稍多时候（一个月中有几次）

4多数时候（一个星期一次）

5几乎每天（一个星期中有几次）

6每天

18. 工作中与病人或同事的密切接触使我感到满足和愉快

0从未有过

1极少数时候(一年中有几次或更少)

2少数时候（一个月一次或更少）

3稍多时候（一个月中有几次）

4多数时候（一个星期一次）

5几乎每天（一个星期中有几次）

6每天

19. 从事医务工作使我体验到自身的价值所在

0从未有过

1极少数时候(一年中有几次或更少)

2少数时候（一个月一次或更少）

3稍多时候（一个月中有几次）

4多数时候（一个星期一次）

5几乎每天（一个星期中有几次）

6每天

20. 我感到自己的情感到了山穷水尽的地步

0从未有过

1极少数时候(一年中有几次或更少)

2少数时候（一个月一次或更少）

3稍多时候（一个月中有几次）

4多数时候（一个星期一次）

5几乎每天（一个星期中有几次）

6每天

21. 我能冷静地处理工作中所遇到的情绪困扰

0从未有过

1极少数时候(一年中有几次或更少)

2少数时候（一个月一次或更少）

3稍多时候（一个月中有几次）

4多数时候（一个星期一次）

5几乎每天（一个星期中有几次）

6每天

22. 我觉得某些病人或家属把他们应面对的问题归咎于我

0从未有过

1极少数时候(一年中有几次或更少)

2少数时候（一个月一次或更少）

3稍多时候（一个月中有几次）

4多数时候（一个星期一次）

5几乎每天（一个星期中有几次）

6每天
