# Supplementary material for: The occupational burnout among medical staff with high workloads after the COVID-19 and its association with anxiety and depression
Source: Front Public Health. 2023 Oct 26;11:1270634. doi: 10.3389/fpubh.2023.1270634 (PMC10639132; doi:10.3389/fpubh.2023.1270634)
Supplement: Supplementary file 2 [file Data_Sheet_2.docx]

**S2 Chinese version of Generalized Anxiety Disorder Questionnaire 7**

1. 感到不安、担心、烦躁或者易怒

0从没有

1有几天

2一天半数

3几乎每天

2. 不能停止或无法控制担心

0从没有

1有几天

2一天半数

3几乎每天

3. 对各种各样的事情担忧过多

0从没有

1有几天

2一天半数

3几乎每天

4. 很紧张，无法放松

0从没有

1有几天

2一天半数

3几乎每天

5. 非常焦躁，以至无法静坐

0从没有

1有几天

2一天半数

3几乎每天

6. 变得很易怒或躁动

0从没有

1有几天

2一天半数

3几乎每天

7. 担忧会有不祥的事情发生

0从没有

1有几天

2一天半数

3几乎每天
