# Supplementary material for: The occupational burnout among medical staff with high workloads after the COVID-19 and its association with anxiety and depression
Source: Front Public Health. 2023 Oct 26;11:1270634. doi: 10.3389/fpubh.2023.1270634 (PMC10639132; doi:10.3389/fpubh.2023.1270634)
Supplement: Supplementary file 3 [file Data_Sheet_3.docx]

**S3** Chinese version of Patient Health Questionnaire 9

1. 对事情没有兴趣

0从没有

1有几天

2一天半数

3几乎每天

2. 感到情绪低下，抑郁，没有希望

0从没有

1有几天

2一天半数

3几乎每天

3. 无法入睡或睡眠时间过长

0从没有

1有几天

2一天半数

3几乎每天

4. 感到疲倦或没有精力

0从没有

1有几天

2一天半数

3几乎每天

5. 没有胃口或狂吃

0从没有

1有几天

2一天半数

3几乎每天

6. 感到内疚或感到自己是失败者或造成家人不成功

0从没有

1有几天

2一天半数

3几乎每天

7. 做事时无法精力集中，如读报或看电视

0从没有

1有几天

2一天半数

3几乎每天

8. 走动或说话相当慢或超出寻常的兴奋和走动

0从没有

1有几天

2一天半数

3几乎每天

9. 想到最好死了算了或自我伤害

0从没有

1有几天

2一天半数

3几乎每天
